# Early life stress and frailty in old age: the Helsinki birth cohort study

**DOI:** 10.1186/s12877-018-0873-5

**Published:** 2018-08-13

**Authors:** M. J. Haapanen, M. M. Perälä, M. K. Salonen, E. Kajantie, M. Simonen, P. Pohjolainen, A. K. Pesonen, K. Räikkönen, J. G. Eriksson, M. B. von Bonsdorff

**Affiliations:** 1 Department of General Practice and Primary Health Care, University of Helsinki and Helsinki University Hospital, PO Box 20, FI-00014 Helsinki, Finland; 2 Folkhälsan Research Center, Helsinki, Finland; 3 Department of Public Health Solutions, Chronic Disease Prevention Unit, National Institute for Health and Welfare, Helsinki, Finland; 4 Hospital for Children and Adolescents, Helsinki University Central Hospital and University of Helsinki, Helsinki, Finland; 5 PEDEGO Research Unit, MRC Oulu, Oulu University Hospital and University of Oulu, Oulu, Finland; 6 Finnish Centre of Excellence in Intersubjectivity in Interaction, University of Helsinki, Helsinki, Finland; 7 Age Institute, Helsinki, Finland; 8 Department of Psychology and Logopedics, University of Helsinki, Helsinki, Finland; 9 Gerontology Research Center, Faculty of Sport and Health Sciences, University of Jyväskylä, Jyväskylä, Finland

**Keywords:** Early life stress, Frailty, Life-course, Natural experiment, Risk factor

## Abstract

**Background:**

Evidence suggests that early life stress (ELS) may extend its effect into adulthood and predispose an individual to adverse health outcomes. We investigated whether wartime parental separation, an indicator of severe ELS, would be associated with frailty in old age.

**Methods:**

Of the 972 participants belonging to the present sub-study of the Helsinki Birth Cohort Study, 117 (12.0%) had been evacuated abroad unaccompanied by their parents in childhood during World War II. Frailty was assessed at a mean age of 71 years according to Fried’s criteria.

**Results:**

Thirteen frail men (4 separated and 9 non-separated) and 20 frail women (2 separated and 18 non-separated) were identified. Compared to the non-separated men, men who had been separated had an increased relative risk ratio (RRR) of frailty (age-adjusted RRR 3.93, 95% CI 1.02, 15.11) that persisted after adjusting for several confounders. No associations were observed among women (RRR 0.62; 95% CI 0.13, 2.94).

**Conclusions:**

These preliminary results suggest that ELS might extend its effects not just into adulthood but also into old age, and secondly, that men may be more vulnerable to the long-term effects of ELS.

## Background

Frailty, the clinical condition that affects several physiological systems predisposing an individual to incomplete recovery from minor changes in health status, is associated with adverse outcomes such as falls, hospitalization, disability, institutionalization and premature mortality [1–3]. Early life stress (ELS) comprises of adverse events occurring within the timeframe of childhood, which may have profound long-term effects on the individual’s health [4–6]. Increasing evidence suggests that ELS might affect the aging process, with known associations on stress physiology, inflammation and telomere length that persist into adulthood [7–9]. Age influences the prevalence of frailty, which rises particularly among those aged 80 years or older [10]. Despite many other known risk factors including depressive symptoms [11], impaired psychological well-being [12] and cognitive ability [13], knowledge on early life influences on frailty is scarce.

Recent evidence suggests the existence of sex differences in responsiveness to ELS [14]. Confronted with stress, both adverse and resilient reactions can occur, with evidence indicating that men may cope more poorly with ELS than women [15]. In a study of 1803 individuals of whom 267 had experienced ELS in the form of wartime separation, no differences in physical and psychosocial functioning scores were observed among women at the mean age of 62 years. However, in that study lower physical and psychosocial functioning scores were observed among the separated men than the non-separated men [16] providing some support for potential gender differences in ELS.

During World War II, approximately 70,000 Finnish children were evacuated unaccompanied by their parents to temporary foster care abroad, mainly to Sweden or Denmark, through Government-aided programs and personal relations to protect the young from the strains of war. The resulting experience of ELS provided the setting for a unique natural experiment, the results of which involving chronic health problems including hypertension, coronary heart disease, type 2 diabetes and depressive symptoms, have been described previously in the Helsinki Birth Cohort Study [17]. The present study explores the association between ELS and frailty according to Fried’s criteria [1] at an average age of 71 years.

## Methods

### Study design

The present study is a sub-study of the Helsinki Birth Cohort Study (HBCS) that includes 8760 individuals born in Helsinki between the years 1934 and 1944. All participants had also visited child welfare clinics in the city at those times and later lived in Finland in 1971 when all Finnish residents were assigned unique personal identification numbers [18]. Random-number tables were used to select individuals for a clinical study. Out of 2902 invited participants, 2003 were examined clinically between 2001 and 2004. The process was repeated for a clinical follow-up between 2011 and 2013. Out of 1404 invited participants, 1094 were examined and 1078 had adequate information on frailty [19]. ELS data was missing for 106 of these cohort members, and they were therefore excluded from the present analyses, resulting in a sample of 972 participants (439 men and 533 women).

### Early life stress

Information on wartime evacuations were obtained from a register in the Finnish National Archives that includes documentation of the 48,628 children sent abroad through the Finnish government. The evacuations and their historical context have been described in detail elsewhere [7]. In addition, approximately over 20,000 more children were evacuated through other channels and therefore questions related to wartime separation were embedded in surveys during the clinical check-ups. In the present study, 117 participants had been sent abroad unaccompanied by their parents. Of these, 99 had information from the register on the duration of the evacuation and 101 on the age at evacuation. Age at separation was coded as: separated before the age of 4 years and at 4 years or older. The duration of the separation was coded as: under 1 year, from 1 to 2 years and 2 years or more. The remaining 855 participants, who had not been evacuated during wartime, served as controls.

### Frailty

Frailty was assessed using five criteria [1] including weight loss, exhaustion, low physical activity, weakness and slowness between 2011 and 2013, as described in previous studies from this cohort [20, 21]. Briefly, recent weight loss was inquired in a questionnaire [22], and those who reported losing 5 kg or more, met the criterion. The criterion of exhaustion was met if the response to the question “How many times during the last week have you felt unusually tired or weak?” was ‘On 3 days or more’. A validated questionnaire [23] was used to assess physical activity. Participants with a total physical activity time of 1 h or less per week met the criterion of low physical activity. Maximal walking speed over a 4.57 m distance was used to assess slowness. The criterion of slowness was met if the participant’s sex- and height-stratified walking speed belonged to the lowest 20%. Weakness was assessed based on the participants’ isometric grip strength of the dominant hand. The criterion of weakness was met if the participant belonged to the lowest 20% of grip strength according to sex-specific quartiles of BMI. Based on the number of met criteria, the cohort members were classified as frail (three or more), pre-frail (one or two) or non-frail (no met criteria).

### Covariates

Data on birth weight was extracted from hospital birth records. Childhood socio-economic status (SES) was estimated based on information about the father’s occupational status available at records held at child welfare and school healthcare clinics. Information on adult SES was obtained from Statistics Finland. The participants were grouped into upper and lower middle class, self-employed and manual workers based on their maximal occupational status attained at 5-year intervals between the years 1970 and 2000. The participants’ height and weight were measured at the clinical examination between the years 2001 and 2004. BMI was calculated as weight in kilograms divided by square of height in meters (kg/m^2^). Questionnaires about health behaviors as smoking (current smoker, former smoker, never smoker) and chronic diseases as hypertension and diabetes were administered at the clinical examination.

### Statistics

Results for continuous values are expressed as means and standard deviations (SD) and were tested using one-way ANOVA analyses. For dichotomous or categorical values, results are expressed as proportions and were tested using cross tabulation. The interaction between sex and wartime separation on frailty was *p* = 0.184. However, we analyzed men and women separately because of earlier findings on the gender differences in the association between ELS and later health outcomes [16]. Multinomial regression analyses were used to study the association between ELS and frailty in old age. Logistic regression analyses were used to study ELS and frailty sub-criteria. The analyses were first adjusted for age and then additionally for birth weight, childhood and adult SES, adulthood BMI, smoking and the prevalence of hypertension and diabetes. Multiple imputations was used to obtain a dataset complete on all main variables and covariates. Imputation was applied to the covariates (for childhood SES *n* = 4, adult BMI *n* = 11, smoking *n* = 6, hypertension *n* = 2 and diabetes *n* = 2; maximum proportion of data missing was 1.1%) and a total of 10 imputed datasets were created. We first performed the analyses using data available for all variables and then using the imputed datasets combining the effect estimates according to Rubin’s rules. We report findings on the imputed data since these results were very similar. The analyses are two-tailed, the level of significance was set at *p* < 0.05 and analyses were carried out with SPSS (IBM SPSS Statistics for Windows, Version 23.0 IBM Corp. Released 2015, Armonk, NY).

## Results

Baseline characteristics of the 972 men and women according to wartime separation status in childhood are presented in Table 1. Apart from birth weight, which was higher among the separated (3.47 kg and 3.41 kg, *p* = 0.006), and hypertension, which was more prevalent among the separated (41.9% and 30.5%, *p* = 0.013), no statistically significant differences were observed between the separated (*n* = 117) and non-separated groups. Most commonly, wartime separation took place after the age of 4 years (mean = 4.2 years) and lasted between 1 to 2 years (mean = 1.6 years).

**Table 1:** Characteristics of the study cohort (*N* = 972)

	Separated (*N* = 117)	Non-separated (*N* = 855)	p^a^
	Mean (SD) or %	Mean (SD) or %	
Sex			0.53
Women (%)	52.1	55.2	
Birth characteristics			
Birth weight (kg)	3.47 (0.48)	3.41 (0.46)	0.006
Childhood socio-economic status			0.32
Upper middle class (%)	14.5	20.4	
Lower middle class (%)	24.0	22.1	
Manual workers (%)	61.5	57.5	
Adulthood characteristics			
Age (years)	72.8 (2.6)	70.5 (2.5)	0.13
Weight (kg)	78.8 (13.8)	76.6 (14.4)	0.55
Height (cm)	168.4 (9.2)	168.6 (9.1)	0.48
BMI (kg/m^2^)	27.8 (4.3)	26.9 (4.5)	0.40
Current smoker (%)	15.4	19.8	0.28
Prevalence of diabetes (%)	7.7	5.4	0.82
Hypertensive (%)	41.9	30.4	0.01
Adult socio-economic status			0.40
Upper middle class (%)	15.4	17.7	
Lower middle class (%)	40.2	45.8	
Self-employed (%)	9.4	8.5	
Manual workers (%)	35.0	28.0	
Age at wartime separation (years)^b^	4.2 (2.2)		
< 4 years (%)	35.9		
≥ 4 years (%)	50.4		
Duration of wartime separation (years)^c^	1.6 (1.0)		
< 1 year (%)	20.5		
1–2 years (%)	42.7		
> 2 years (%)	21.4		

*Note.* BMI = body mass index

^a^Difference between the separated and the non-separated

^b^Available for 86.3% of the separated

^c^Available for 84.6% of the separated

Of the 61 women and 56 men experiencing wartime separation, 2 frail women and 4 frail men were identified, respectively. The prevalence of frailty was 5.1% for the separated and 3.2% for the non-separated cohort members, and it was higher among the separated men compared to non-separated men (7.1% and 2.3%, *p* = 0.048, Table 2). No significant differences in the individual frailty sub-criteria were observed between the separated and non-separated cohort members.

**Table 2:** The prevalence of frailty and its sub-criteria according to wartime separation status for the entire cohort and separately for men and women

	Separated (*N* = 117)	Non-separated (*N* = 855)	p^a^
Frailty classification			0.31
Non-frail (%)	51.3	57.5	
Men (%)	50.0	58.0	0.26
Women (%)	52.5	57.2	0.48
Pre-frail (%)	43.6	39.3	
Men (%)	42.9	39.7	0.65
Women (%)	44.3	39.0	0.43
Frail (%)	5.1 (*N* = 6)	3.2 (*N* = 27)	
Men (%)	7.1 (*N* = 4)	2.3 (*N* = 9)	0.048
Women (%)	3.3 (*N* = 2)	3.8 (*N* = 18)	0.84
Frailty criteria			
Weight loss (%)	3.4	5.6	0.33
Men (%)	7.3	6.1	0.80
Women (%)	4.9	5.5	0.61
Exhaustion (%)	9.4	6.9	0.30
Men (%)	3.7	5.2	0.76
Women (%)	14.8	8.3	0.097
Low physical activity (%)	8.5	9.8	0.66
Men (%)	5.4	7.8	0.60
Women (%)	11.5	11.4	0.99
Slowness (%)	24.8	19.1	0.15
Men (%)	28.6	19.1	0.098
Women (%)	21.3	19.1	0.68
Weakness (%)	22.2	19.3	0.36
Men (%)	28.6	18.1	0.065
Women (%)	17.5	20.4	0.61

^a^Difference between the separated and the non-separated

Compared to the non-separated men, those separated in childhood had an increased relative risk ratio (RRR) of frailty (age-adjusted RRR 3.93, 95% CI 1.02, 15.11) relative to non-frailty (Table 3). The association remained significant after adjusting further for birth weight, childhood and adulthood SES, adulthood BMI, smoking as well as the prevalence of hypertension and diabetes, RRR 5.18 (95% CI 1.16 to 23.17). When the number of met frailty criteria (0 to 5) was implemented as a continuous variable, a similar trend for the men was observed (*p* = 0.073). No associations were observed between wartime separation status and frailty in women.

**Table 3:** Relative risk ratios (RRR’s) and 95% confidence intervals (CI’s) for frailty among the men and women who were separated in childhood compared to the non-separated

	Age-adjusted		Adjusted^a^	
	RRR (95% CI)	p	RRR (95% CI)	p
Non-frail	Ref.		Ref.	
Pre-frail				
Men	1.18 (0.64 to 2.17)	0.60	1.17 (0.62 to 2.22)	0.63
Women	0.90 (0.51 to 1.60)	0.71	0.77 (0.42 to 1.39)	0.38
Frail				
Men	3.93 (1.02 to 15.11)	0.046	5.18 (1.16 to 23.17)	0.031
Women	0.62 (0.13 to 2.94)	0.55	0.58 (0.26 to 1.31)	0.50

^a^Adjusted for age, birth weight, socio-economic status in childhood and adulthood, adulthood BMI, smoking and the prevalence of hypertension and diabetes

The associations between age at and duration of separation and frailty were studied further among the men in the cohort (Table 4). Compared to the non-separated men, those men who had been separated under the age of 4 years had an RRR of 5.58 (95% CI 1.36, 22.93) of frailty. Additional adjustments for confounders did not attenuate the association, RRR 6.96 (95% CI 1.28, 37.72). Further, those men who had been separated for more than 2 years had a RRR of 10.27 (95% CI 1.71, 61.68) of frailty compared to the non-separated. The association remained significant after further adjustments, RRR 12.87 (95% CI 1.29, 128.38). No associations between age at and duration of the separation and frailty were observed among women (data not shown).

**Table 4:** Relative risk ratios (RRR’s) and 95% confidence intervals (CI’s) of frailty in men according to age at and duration of separation among the separated compared to the non-separated

	Non-frail (ref.)	Pre-frail				Frail			
		Age-adjusted	p	Adjusted^a^	p	Age-adjusted	p	Adjusted^a^	p
Age at separation (years)									
Non-separated		Ref.		Ref.		Ref.		Ref.	
< 4		0.94 (0.39 to 2.25)	0.89	1.06 (0.42 to 2.68)	0.91	5.58 (1.36 to 22.93)	0.017	6.96 (1.28 to 37.72)	0.024
≥ 4		0.94 (0.35 to 2.52)	0.90	0.95 (0.34 to 2.64)	0.93	No data		No data	
Duration of separation (years)									
Non-separated		Ref.		Ref.		Ref.		Ref.	
≤ 1		0.61 (0.18 to 2.09)	0.43	0.49 (0.14 to 1.74)	0.27	No data		No data	
> 1 and ≤ 2		1.05 (0.41 to 2.71)	0.91	1.21 (0.44 to 3.31)	0.71	2.66 (0.28 to 25.15)	0.39	3.26 (0.24 to 44.44)	0.37
> 2		1.02 (0.27 to 3.87)	0.98	1.21 (0.30 to 4.96)	0.79	10.27 (1.71 to 61.68)	0.011	12.87 (1.29 to 128.38)	0.029

^a^Adjusted for age, birth weight, childhood and adulthood socio-economic status, adulthood BMI, smoking and the prevalence of hypertension and diabetes

The individual frailty sub-criterion of *weakness* seemed to be driving the association between ELS and frailty: compared to the non-separated women, women who had been separated in childhood had an adjusted RRR of 0.43 (95% CI 0.20, 0.95; *p* = 0.037) for meeting the criterion of *weakness* (data not shown). For men, a borderline significant association (*p* = 0.06) indicated that the association between wartime separation status and *weakness* in old age was opposite to that observed for women, RRR 2.01 (95% 0.97, 4.15). No associations between wartime separation and other frailty sub-criteria were observed (data not shown).

## Discussion

We found preliminary evidence that separation from both parents due to wartime evacuation, an indication of ELS, was associated with an increased risk of frailty in men at a mean age of 71 years. No associations between ELS and frailty were observed among women. The association was strongest for those men who were separated at an early age and who were separated for the longest time period. Further, of the individual components included in the Fried criteria for frailty, separation status was most strongly associated with *weakness* showing an increased risk in men but a decreased risk in women.

This is to our knowledge the first study that focuses upon the association between ELS and frailty in old age. ELS has previously been linked with health behaviours that may increase later chronic disease risk [6]. However, we found no statistically significant differences in SES or smoking between the separated and the non-separated. The observed prevalence of frailty was higher in the separated group (5.1%) than in the non-separated group (3.2%) with no significant gender differences, however lower than that reported in other studies with participants of similar age [24, 25]. Findings from the present study are supported by a previous finding linking wartime separation with lower physical and psychosocial functioning in men [16].

Experiments in animals suggest a number of potential biological mechanisms [26]. These include those involving the hypothalamus-pituitary-adrenal (HPA) axis and consequently cortisol metabolism and stress reactivity in later life [27]. We have shown in a subsample of the HBCS that individuals who were exposed to wartime separation in early life had increased salivary cortisol and plasma ACTH concentrations six decades later compared with those who had not been separated. Separated men did not differ in terms of baseline plasma cortisol and ACTH concentrations as seen in separated women, but they reacted more powerfully in a stress test showing significantly higher salivary cortisol concentrations and AUC increments compared to the non-separated men [7]. This might give insight into the sex difference observed in the present study. Developmental differences between men and women might also explain the gender difference observed in the study [15]. Interactions between the new foster environment and the evacuees’ genetic factors may have resulted in epigenetic changes that predispose at-risk individuals to the harmful effects of ELS. Further, it is also possible that ELS by itself could induce changes in gene expression. [28]

Inflammation could also mediate the association between ELS and frailty in old age. Higher levels of inflammatory markers have been measured among adults who had experienced adversities early in life [8]. In the social context of the study, wartime separation may have impeded the development of supportive close relations vital for buffering against inflammatory processes [29]. Besides frailty, inflammation is recognized as a risk factor for several other chronic illnesses [30]. ELS has been linked with hypertension, coronary heart disease, type 2 diabetes and depressive disorders in adulthood, with no sex difference similar to that observed in the current study [31–34]. Simultaneous presence of several chronic conditions may further predispose an individual to disturbances in homeostasis that are characteristic of frailty [35].

A key strength of this well-characterized longitudinal clinical study cohort is the objective definition of wartime separation status. Information on age at and duration of the separation was extracted from government registers. We were able to investigate the long-term association between ELS and frailty in old age with a particularly long follow-up. Data on birth anthropometry and SES were extracted from birth records and national registers. Frailty was defined according to Fried [1] using standardized methods.

However, caution should be taken when interpreting the results. A major limitation of the present study is the small number of separated frail men (*n* = 4) and women (*n* = 2) which limits our ability in estimating the strength of the found associations. The possibility of a chance finding cannot be ruled out and therefore the associations described in this study should be considered preliminary and need to be replicated. Not all evacuations were executed through the Finnish government and participants may have been misclassified as non-separated despite having been separated through personal relations, and to identify these individuals, questionnaires about wartime separation were administered at the clinical visits. There was no information available on the quality of foster care or living conditions during the separation period. During the long follow-up we might have missed cohort members who were in poor health, which could consequently lead to selective survival and an underrepresentation of frailty in the present study. The cohort consists of participants born in Helsinki between the years 1934–1944 and who at that time attended voluntary child welfare clinics in the city. Because attendance to the clinics was voluntary, the study population may not have been representative of that in the city. Although separation during World War II created unique conditions in the present study, today millions of refugees experience maltreatment and poverty of which the long-term consequences are still unknown.

## Conclusions

Results from the current study provide preliminary evidence that the consequences of ELS may persist into old age. The association between ELS and frailty was restricted to men and was stronger among those men who were separated at an earlier age and for a longer period of time. The results are preliminary and are to be confirmed in larger samples with more separated frail individuals.
